# An Enhanced Vacuum-Assisted Resin Transfer Molding Process and Its Pressure Effect on Resin Infusion Behavior and Composite Material Performance

**DOI:** 10.3390/polym16101386

**Published:** 2024-05-13

**Authors:** Rulin Shen, Taizhi Liu, Hehua Liu, Xiangfu Zou, Yanling Gong, Haibo Guo

**Affiliations:** College of Mechanical and Electrical Engineering, Central South University, Changsha 410083, China; shenrl@csu.edu.cn (R.S.); csu2337ltz@csu.edu.cn (T.L.); aixin_lang@163.com (H.L.); gongyanling@csu.edu.cn (Y.G.);

**Keywords:** VARTM process, resin infusion behavior, composite laminates, mechanical property, fiber volume fraction

## Abstract

In this paper, an enhanced VARTM process is proposed and its pressure effect on resin infusion behavior and composite material performance is studied to reveal the control mechanism of the fiber volume fraction and void content. The molding is vacuumized during the resin injection stage while it is pressurized during the mold filling and curing stages via a VARTM pressure control system designed in this paper. Theoretical calculations and simulation methods are used to reveal the resin’s in-plane, transverse, and three-dimensional flow patterns in multi-layer media. For typical thin-walled components, the infiltration behavior of resin in isotropic porous media is studied, elucidating the control mechanisms of fiber volume fraction and void content. The experiments demonstrate that the enhanced VARTM process significantly improves mold filling efficiency and composite’s performance. Compared to the regular VARTM process, the panel thickness is reduced by 4% from 1.7 mm, the average tensile strength is increased by 7.3% to 760 MPa, the average flexural strength remains at approximately 720 MPa, porosity is decreased from 1.5% to below 1%, and the fiber volume fraction is increased from 55% to 62%.

## 1. Introduction

Vacuum-assisted resin transfer molding (VARTM) technology is a cost-effective and efficient composite molding process, which has been widely used in many fields such as aerospace, marine vessels, wind power, energy, automotive, and sports. However, compared to pre-impregnated molding processes, the VARTM process still exhibits certain disparities in fiber volume fraction and porosity, and its material mechanical properties are slightly inferior. Challenges in the formation of large, complex components persist, including uneven resin flow, extended mold filling times, and dimensional accuracy issues. Improving the VARTM process to increase the resin wetting speed, improve the resin wetting effect, increase the fiber volume fraction, and reduce the void content is of great significance to enhance the mechanical properties of the materials.

The VARTM process belongs to the Liquid Composite Molding (LCM) family, and its inherent limitations are primarily attributed to issues related to resin flow. Various factors such as mold dimensions and structures, fiber preform design, type, layering, stacking sequence, pressure gradient distribution, and more all impact the flow of resin within the VARTM process. Therefore, improvements in the VARTM process primarily focus on these aspects and can be summarized into three main methods: (i) heating the resin to reduce its viscosity [1,2,3,4,5,6]; (ii) adjusting the pressure distribution on the mold surface [7,8,9,10,11]; and (iii) making the fiber prefabricated parts loose to increase the permeability [12,13,14,15,16,17,18,19].

Temperature has a significant impact on the viscosity of resin. Generally, resin viscosity decreases as temperature rises, with lower viscosity making the resin flow more easily. Therefore, heating the resin can reduce its viscosity, enhancing its flowability. Heating the resin can also reduce the resistance during resin flow, aiding in bubble removal since thermal energy facilitates bubble diffusion and escape. Heating the resin can be performed directly or indirectly through heating the metal mold. Directly heating the resin is performed before resin injection, and as the resin temperature quickly drops during the injection process, it is suitable for manufacturing small components, where resin injection can be completed rapidly. Indirectly heating the resin involves using an electrical heating device to continuously heat the mold [1,2,3]. This method ensures more even resin heating, reducing the impact of temperature gradients on the quality of the formed product, thereby maintaining consistent molding quality. However, it requires a metal mold and has a relatively slow heating rate.

One major drawback of heating the resin is that it enhances the activity of the resin, accelerates the curing speed, and potentially causes premature curing reactions. This is not suitable for resins with short activation times or for larger-sized components [4,5,6].

Adjusting the pressure distribution on the mold surface to enhance the resin flow speed is a simple and rapid method. Increasing the number of resin injection and vacuum ports on the mold or laying resin channels on the surface of the fiber preform, reducing the distance between the inlet and outlet, and increasing the pressure gradient can accelerate resin flow [7,8]. However, this method results in a significant consumption of experimental materials and resin, leading to higher costs, and the fiber infiltration effect may not be very satisfactory. As a result, some researchers have proposed the use of reusable pressure distributors. By applying different vacuum pressures to two sealed chambers, they control the pressure difference between the two chambers in a pulsating manner. This promotes in-plane resin flow through the fiber preform and achieves excellent resin impregnation into the fibers [9,10,11]. And some researchers have used the creation of a certain vacuum environment above the mold to regulate the pressure distribution on the surface of the mold, thus changing the permeability of the fiber preform and improving the infiltration of the resin [12,13,14,15,16].

Enhancing the permeability of fiber preforms can fundamentally address the issue of slow resin infiltration. However, the vacuum pressure generated by the VARTM process can compact the fiber layers, reducing their permeability. Hence, some scholars have proposed the dual vacuum bag method, created two separate sealed chambers, and controlled the vacuum pressure in these inner and outer chambers to allow for the fiber preforms to relax and improve their permeability [12,13]. Others have used external rigid vacuum chambers above the mold to extract a certain degree of vacuum, thereby increasing the permeability of the fiber preforms to improve resin flow patterns [2,14,15,16,17,18,19].

Resin-infusion molding is a variation of the VARTM process that involves the use of a distribution medium. It increases resin flow speed by enhancing permeability [14,20,21]. However, it does not directly increase the permeability of the fiber preform. Instead, it involves placing a high-permeability medium on top of the fiber layers to guide resin flow into less permeable regions. While this method addresses the issues of slow and uneven resin flow, it does not resolve the problem of insufficient fiber impregnation. The fiber layers beneath the distribution medium still cannot be adequately impregnated in a timely manner, leading to unsaturated resin flow within the lower fiber layers and the formation of numerous bubbles. Consequently, this results in significant variations in porosity along the thickness direction of the laminated panel [22,23]. In addition, placement and removal of the distribution medium not only increases the overall process time, consumable costs, and waste of non-recyclable materials but also requires additional labor. Distribution mediums often result in textured laminate surfaces, which may degrade the quality of the laminate.

This paper introduces an enhanced VARTM process, where a bi-directional adjustable pressure unit is designed to vacuumize or pressurize the molding. The molding is vacuumized during the resin injection stage while it is pressurized during the mold filling and curing stages. Differing from existing enhancement methods, this approach achieves a dual purpose within the VARTM process. First, during the resin injection stage, maintaining a similarly high vacuum level inside both the vacuum bag and the pressure chamber aids in the relaxation of the fiber preforms, allowing the resin to be quickly injected into the vacuum bag. Second, during the mold filling and curing stages, the application of additional pressure within the pressure chamber promotes the flow of resin under vacuum conditions within the vacuum bag, improving fiber impregnation while simultaneously allowing the laminate to be thinner, thereby increasing the fiber volume fraction.

In this study, theoretical calculations were first carried out to analytically investigate the in-plane, transverse, and three-dimensional resin flow behaviors in multi-layer media, and reveal the flow patterns. Then, the flow patterns of the resin were further investigated using simulation methods, as well as the flow behavior of the resin in planar isotropic porous media for typical thin-walled components, and the control mechanisms of fiber volume fraction and porosity were elucidated. Finally, molding experiments were conducted to validate resin flow patterns and mechanisms for refining molding parameters.

The structure of this paper can be summarized as follows. In Section 2, the flow patterns of resin under pressure are investigated by theoretical analysis and simulation methods. Section 3 introduces the experimental methods of the enhanced VARTM process. In Section 4, we compare and analyze the experimental results. Finally, Section 5 summarizes the conclusions drawn from the study.

## 2. Resin Flow Patterns under Pressure

The permeability coefficients of a single layer of fiber reinforcement in all directions of the plane can be considered to be the same, but the actual composite products are often made of multiple layers of different fiber reinforcements stacked on top of each other, such as carbon fiber, glass fiber, Kevlar fiber, and so on. Therefore, in the vertical direction, the permeability of different fiber reinforcement or interlayer materials is obviously different [24,25]. When the product is thicker, the permeability in the vertical direction has an effect on the flow of resin during the mold filling process. The resin in multi-layer fibers generates both in-plane (in the horizontal direction) and transverse (in the vertical direction) flow processes under external pressure. The study of resin infiltration and mold filling behavior under the effect of an applied pressure field reveals the infiltration behavior of resin in different directions under the effect of a pressure field, which can provide the theoretical basis and technical support for the design and optimization of VARTM process parameters.

### 2.1. In-Plane Infiltration Characteristics of Resin within the Fiber Layers

**Figure 1:**
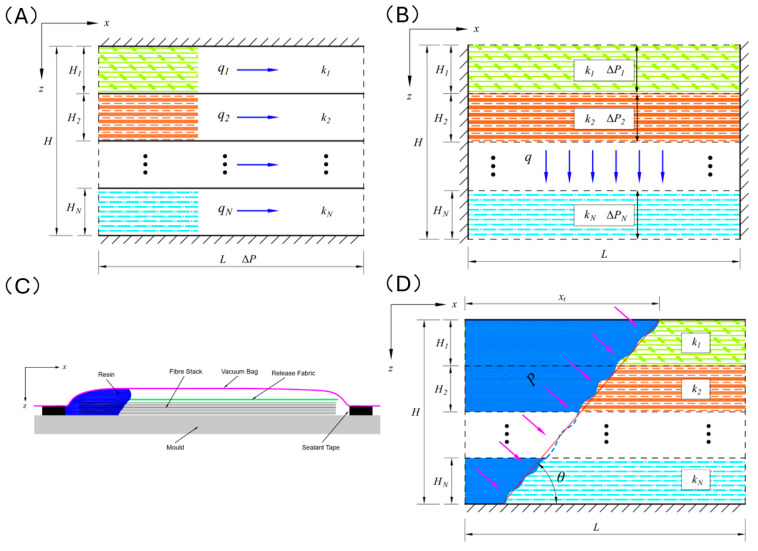
(**A**) Schematic diagram of the in-plane flow of resin within the fiber layers. (**B**) Schematic diagram of the transverse flow of resin within and between the fiber layers. (**C**) Initial morphology of the resin after injection into the vacuum bag. (**D**) Schematic diagram of simultaneous in-plane and transverse flow of resin within and between the fiber layers. (The arrows in the figure represent the direction of resin flow, and “P” represents the pressure exerted on the resin flow. Other letters are explained in the main text).

As the permeability of each fiber layer varies, the vertical cross-section of the multi-layer fibers is a kind of non-homogeneous isotropic porous medium. When the resin is injected from one end and exits from the other end under pressure parallel to the fiber level, the pressure gradient in the resin infiltration region is uniformly distributed, with an equal pressure gradient in each layer. The effects of the resin’s own gravity and capillary forces are neglected as the VARTM process generates a driving pressure of one atmosphere, which is much larger than the capillary force between the fibers and the resin [26,27,28]. A vertical cross-sectional region of a fully infiltrated fiber layer is shown in Figure 1A.

Based on Darcy’s law, if we calculate the flow rate per unit width *q_i_* within each layer of fibers, the total flow rate *q* equals the sum of the flow rate per unit width in each layer:

(1) $$q=\sum_{i=1}^{N}q_{i},H=\sum_{i=1}^{N}H_{i},q_{i}=\frac{k_{i}H_{i}}{\mu }\frac{\Delta P}{L}$$

where $k_{i}$, $H_{i}$, and $q_{i}$ denote the permeability (m^2^), thickness (m), and flow rate per unit width (m^2^/s) of layer *i*, ΔP is the pressure difference between the two ends of the fiber layer (Pa), L is the infiltration path (m), N is the number of fiber layers, and μ is the dynamic viscosity of the resin (N·s/m^2^ or Pa·s).

The in-plane infiltration velocity of the resin within each fiber layer is the following:

(2) $$u_{i}=\frac{q_{i}}{H_{i}}=\frac{k_{i}\Delta P}{\mu L}$$

The pressure gradient ΔP/L remains constant within each layer, therefore, we obtain the following:

(3) $$q=\sum_{i=1}^{N}q_{i}=\sum_{i=1}^{N}\frac{k_{i}}{\mu }H_{i}\frac{\Delta P}{L}=\frac{\Delta P}{\mu L}\sum_{i=1}^{N}k_{i}H_{i}$$

If each layer of fiber is thin, and there are many layers of fibers, it can be assumed that the permeability varies continuously along the vertical direction. Therefore, the total flow of resin flowing through the thickness H of the entire fiber layer, parallel to the plane of the layers, is given by the following:

(4) $$q=\frac{\Delta P}{\mu L}\int_{0}^{H}k(z)dz$$

The integral form of the in-plane equivalent infiltration velocity is given by the following:

(5) $$\bar{u}=\frac{{\bar{q}}_{h}}{H}=\frac{\Delta P}{\mu HL}\int_{0}^{H}k(z)dz$$

### 2.2. Transverse Infiltration Characteristics of Resin within and between the Fiber Layers

When resin is subjected to external pressure and infiltrates into multiple layers of fibers, in a localized area, it can be considered that the resin is uniformly distributed on the surfaces of multiple layers of fibers. Under the influence of external pressure (when using the enhanced VARTM process, the resin’s own gravity and capillary forces are much smaller than the applied pressure, so they can be similarly neglected), the resin infiltrates downward through the gaps between fiber bundles and within the bundles themselves. Consider an infiltration region perpendicular to the plane of the fiber layers, as shown in Figure 1B.

The flow path aligns with the thickness of the fiber layers, as shown in Figure 1B. Therefore, the flow rate through each layer is equal to the total flow rate, and the pressure varies within each layer. The total pressure is the sum of the pressure in each layer, which can be determined using Darcy’s law as follows:

(6) $$q=\frac{L\cdot \Delta P}{\mu \sum_{i=1}^{N}\frac{H_{i}}{k_{i}}}$$

It can be deduced that the equivalent permeability of the multi-layer fibers in the vertical direction ${\bar{k}}_{v}$ can be expressed as the following:

(7) $${\bar{k}}_{v}=\frac{H}{\sum_{i=1}^{N}\frac{H_{i}}{k_{i}}}$$

Similarly, if each layer of fiber is thin, and there are many layers of fibers, it can be assumed that the permeability varies continuously along the vertical direction. Therefore, the integral expression for the effective permeability is given by the following similarly:

(8) $${\bar{k}}_{v}=\frac{H}{\int_{0}^{H}\frac{1}{k(z)}dz}$$

The integral form of the transverse equivalent infiltration velocity is given by the following:

(9) $$\bar{w}=\frac{q_{v}}{L}=\frac{\Delta P}{\mu \int_{0}^{H}\frac{1}{k(z)}dz}$$

### 2.3. Three-Dimensional Infiltration Characteristics of Resin within and between the Fiber Layers

In the enhanced VARTM process, when resin is injected into the mold under atmospheric pressure, it tends to accumulate on the surface of the fiber layers near the injection point. Then, under the influence of the applied pressure, it infiltrates both outward and downward, as shown in Figure 1C. Therefore, in the actual process, resin infiltration within the fiber layers occurs both in-plane and transversely [29,30], as shown in Figure 1D. The resin flow front within the vertical cross-section is approximately regarded as a straight line (due to the different permeability of each layer, in fact, the resin flow front should be a fold line), then the rest of the angle follows the horizontal plane θ.

**Figure 2:**
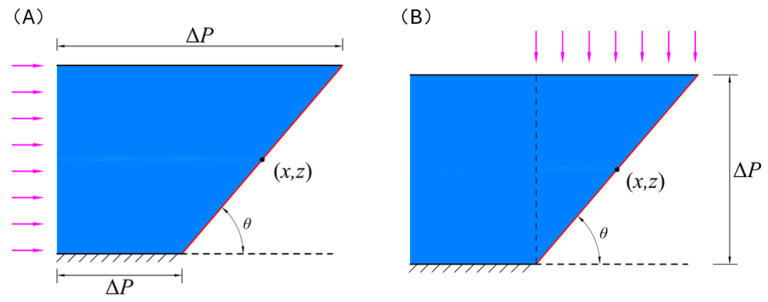
(**A**) Schematic diagram of horizontal forces on the flow front of the resin. (**B**) Schematic diagram of vertical force on the flow front of the resin (The arrows in the figure indicate the direction of resin flow).

In the VARTM process, when the resin has not completely infiltrated the fiber layers, the horizontal pressure gradient varies along the penetration distance. The absolute pressure at the resin injection inlet always remains at atmospheric pressure, and the absolute pressure at the leading edge of the resin flow is always 0. Therefore, within a vertical cross-section, at any point (x,z) on the leading edge of resin flow, as shown in Figure 2A, the pressure gradient in the horizontal direction is a function of the in-plane penetration distance $x_{t}$ and can be expressed as follows:

(10) $$J_{x}=\frac{\Delta P}{x}=\frac{\Delta P}{x_{t}-z\cot\theta }$$

In the already infiltrated region, the pressures in the normal direction on the top surface, bottom surface, and the resin injection inlet end face are all at atmospheric pressure, thus having no relative pressure. Therefore, at any point (x,z) on the leading edge of resin flow as shown in Figure 2B, the pressure gradient in the vertical direction is a function of the transverse penetration distance $z_{t}$ and can be expressed as follows:

(11) $$J_{z}=\frac{\Delta P}{z_{t}}=\frac{\Delta P}{z}$$

Then, the total equivalent flow in the horizontal direction is given by the following:

(12) $${\bar{q}}_{h}=\frac{\Delta P}{\mu }\int_{0}^{H}\frac{k(z)}{x_{t}-z\cot\theta }dz$$

The in-plane equivalent infiltration velocity is given by the following:

(13) $$\bar{u}=\frac{{\bar{q}}_{h}}{H}=\frac{\Delta P}{\mu H}\int_{0}^{H}\frac{k(z)}{x_{t}-z\cot\theta }dz$$

The total equivalent flow in the vertical direction is given by the following:

(14) $${\bar{q}}_{v}=\frac{\Delta P}{\mu }\int_{0}^{H}\frac{k(z)}{z}dz$$

The transverse equivalent infiltration velocity is given by the following:

(15) $$\bar{w}=\frac{{\bar{q}}_{v}}{H\cot\theta }=\frac{\Delta P}{\mu H\cot\theta }\int_{0}^{H}\frac{k(z)}{z}dz$$

According to the principle of vector addition, the equivalent velocity in the normal direction of the resin flow front is given by the following:

(16) $${\bar{v}}_{s}={\left({\bar{u}}^{2}+{\bar{w}}^{2}\right)}^{1/2}=\frac{\Delta P}{\mu H}{\left[{\left(\int_{0}^{H}\frac{k(z)}{x_{t}-z\cot\theta }dz\right)}^{2}+{\left(\frac{1}{\cot\theta }\int_{0}^{H}\frac{k(z)}{z}dz\right)}^{2}\right]}^{1/2}$$

### 2.4. Simulation of Transverse Infiltration of Resin within and between Multi-Layer Fiber Layers

To investigate the flow process of resin within and between fiber layers under the enhanced VARTM process, simulations were conducted to examine the flow patterns, infiltration velocities, and volume fractions (i.e., fiber layer saturation) of resin at the microscopic level within and between the fiber layers. COMSOL Multiphysics, the flagship product of COMSOL Inc. (Stockholm, Sweden), is a powerful multiphysics field coupling analysis software equipped with various built-in physical field modules. Among them, the “Fluid Flow” module incorporates numerous sub-interfaces, such as Darcy’s Law, Richards’ equation, multiphase flow in porous media, and free and porous media flows, making it highly convenient and efficient for simulating percolation problems in porous media. This paper adopts the porous media infiltration module of COMSOL 6.0 software to simulate the flow process of resin within and between the fiber layers.

**Figure 3:**
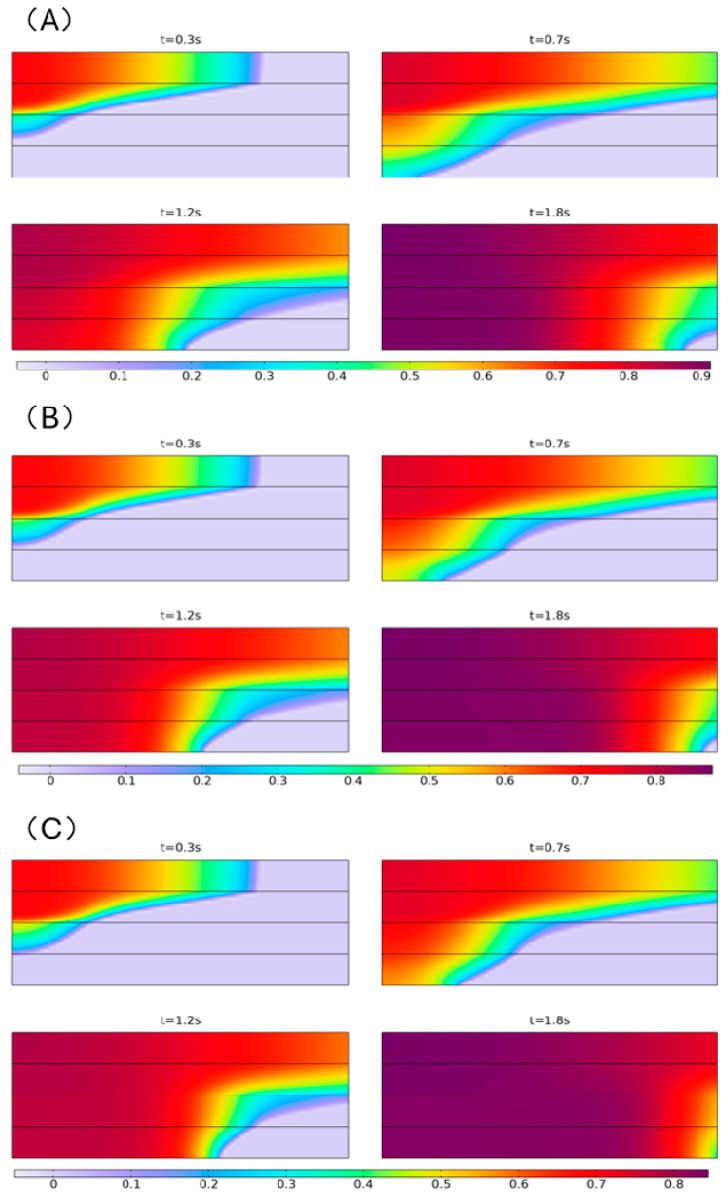
Resin flow states within and between fiber layers at 0.8 bar (**A**), 1.2 bar (**B**), and 1.6 bar (**C**) external pressure.

The flow pattern, infiltration velocities, and volume fractions of the resin in four fiber layers were investigated at three applied pressures of 0.8 bar, 1.2 bar, and 1.6 bar. To maintain consistency with the experimental conditions, the vacuum level within the vacuum bag was set to −0.92 bar. By using an electronic scale and a high-speed camera, the functional relationship between resin flow rate and time can be easily obtained in the experiment. Consequently, the inlet boundary condition was set as the mass flux of the resin, with a magnitude of 3.2 kg/(m^2^·s). In the initial stage of the enhanced VARTM process, resin accumulates above the fiber layers, and under the influence of external pressure, there is a simultaneous flow trend both in-plane (in the horizontal direction) and transversely (in the vertical direction). Therefore, the inlet was set at the left end of the uppermost fiber layer, and the outlet was set at the right end of all fiber layers. The model size is 0.8 mm × 2 mm (each layer 0.2 mm thick). Due to the relatively small model size and to avoid the influence of the woven structure on the flow front, each fiber layer has been homogenized. The simulation duration is 2 s, and the simulation results are shown in Figure 3.

From the simulation results, it can be observed that at the same moment under different external pressure conditions, the morphology of the resin flow front within the fiber layers is almost identical, with slight variations in the position of the flow front. As the external pressure increases, the resin flows faster, and simultaneously, the resin volume fraction becomes lower.

At the initial moment, the resin has a flux only in the in-plane direction, so the infiltration speed in the first layer of fiber is extremely fast. Subsequently, the resin begins to infiltrate downward due to pressure differentials. In each layer, a certain angle forms between the resin flow front and the horizontal plane, resulting in a folded line shape resin flow front. As the distance from the inlet increases, the angle gradually becomes larger.

The legend in the figure represents the volume fraction of resin within the fiber layers, indicating the saturation level of the fiber layers. It can be observed that during the enhanced VARTM process, the maximum value of the resin’s volume fraction within the fiber layers gradually increases with time. This indicates that the resin flow front is in an unsaturated flow state, where the resin is insufficient to adequately displace the air between fibers, resulting in incomplete penetration and occupation of voids within the reinforcing material. The reason for this is that the permeability within the fiber bundles is very low, significantly hindering the flow of resin. However, the inter-bundle pores offer little resistance to resin flow. As a result, there is a velocity difference within and between the fiber bundles, leading to the formation of numerous air bubbles.

On the other hand, the pressure difference between the inlet and outlet ends is the fundamental reason for resin flow. Therefore, when the pressure difference is greater, the resin flows faster, and the phenomenon of unsaturated flow becomes more pronounced.

### 2.5. Simulation of In-Plane Mold Filling Infiltration in Laminated Panels

On a macroscopic scale, the thickness of the fiber layers is neglected, and the fiber layers are treated as planar isotropic porous media. A two-dimensional planar model with dimensions identical to the experimental fiber reinforcement size of 250 × 250 mm was established. Similarly, the vacuum level within the vacuum bag was set to −0.92 bar, consistent with the experimental conditions. According to experimental observations, at the initial moment of pressurizing the pressure vessel, the resin injected into the vacuum bag has already infiltrated a semi-circular area with a radius of approximately 50 mm. The circular vacuum port has a diameter of 10 mm. These two regions are excluded from the geometric modeling.

**Figure 4:**
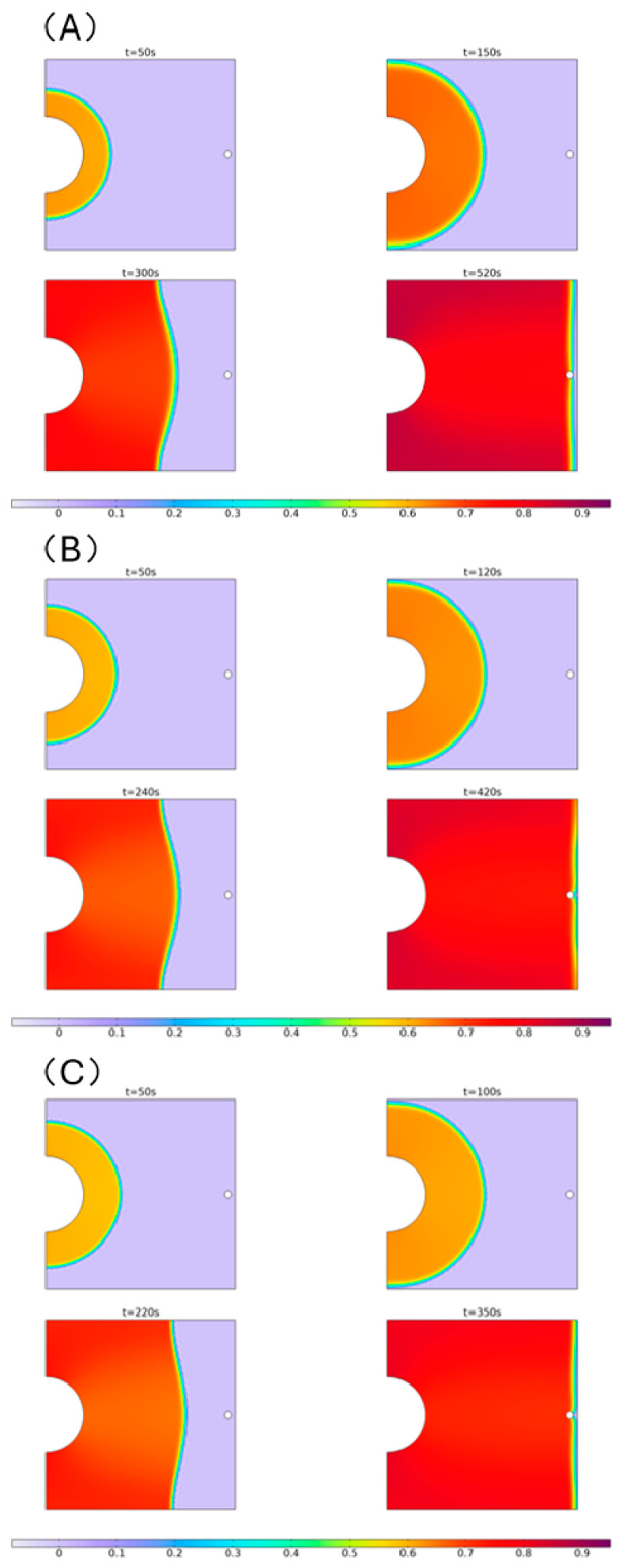
Simulation schematic of mold filling processes of laminate under 0.8 bar (**A**), 1.2 bar (**B**), and 1.6 bar (**C**) external pressure.

This section focuses solely on simulating and analyzing the resin flow process under different pressure conditions in the enhanced VARTM process. It does not include simulation and analysis of the resin flow process under the regular VARTM process (because the flow fronts are quite similar, only with differences in mold filling times). The simulation results are presented in Figure 4.

The simulation results from the mold filling process show that there is almost no significant difference in the macroscopic flow patterns of the resin under the three different pressures. Before the resin flow reaches the two right-angles at the left end, the leading edge of the flow infiltrates and spreads forward in an arc shape. After passing the right-angles, the resin contacts the boundary and ceases to penetrate outwards. Therefore, it rapidly converges at the boundary, forming a “bow”-shaped leading edge of the flow. The curvature of the leading edge decreases continuously with the increasing penetration distance, and finally, it approximately forms a straight line when reaching the extraction port.

**Figure 5:**
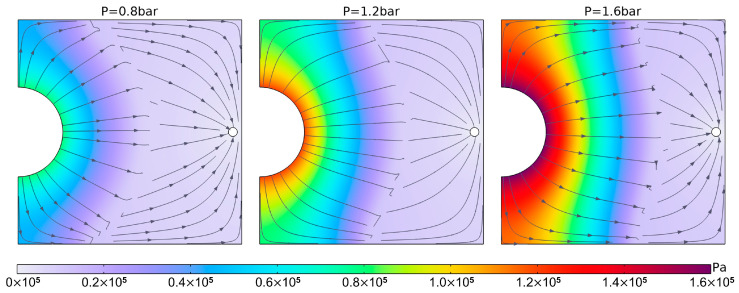
Flow lines of pressure and velocity fields at three different pressures at t = 200 s.

Under three different pressures, there is a noticeable difference in the resin flow rate, with the resin flowing faster as the applied pressure increases. This is reflected in the gradual reduction in the mold filling time with increasing pressure. Figure 5 illustrates the pressure fields and velocity field streamlines of resin and air at t = 200 s under these three different pressures. The Figure 5 shows that under all pressure conditions, the distribution of the pressure field closely aligns with the resin infiltration regions. Especially, within the areas that have already been infiltrated, the pressure gradient is evenly distributed, while in the non-infiltrated regions, the pressure remains relatively at 0. At the leading edge of resin flow, the velocity field streamlines exhibit discontinuities. The already infiltrated region (where pressure is evenly distributed) corresponds to the resin, while the non-infiltrated region (where pressure is 0) corresponds to air. The distribution of streamlines in both regions shows strong regularity.

## 3. Experimental Methods

### 3.1. Experimental Apparatus

**Figure 6:**
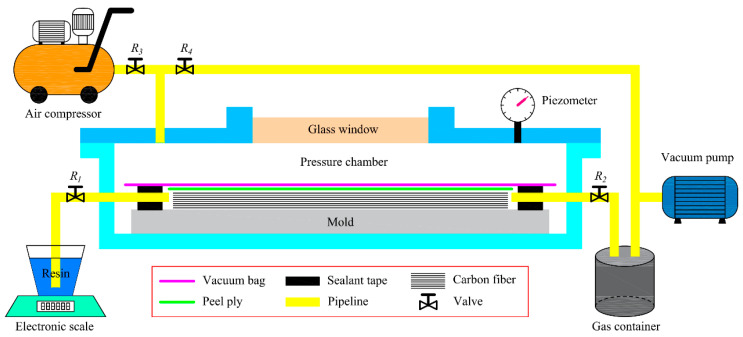
Schematic diagram of the enhanced device-based VARTM process.

In this paper, a detachable pressure chamber is designed to easily adjust the pressure during the resin injection and curing stages to achieve an enhanced VARTM process, as shown in Figure 6. The mold is placed into the pressure chamber together with its surface fiber layer, release cloth and vacuum bag, etc. The resin injection and pumping pipes are connected to the external resin container and air storage tank through the through holes in the wall plate of the pressure chamber, and the gap between the pipes and the mounting holes is sealed with RTM adhesive. The pressure chamber is fitted with a three-way pipe, one end of which is connected to a vacuum pump to provide negative pressure to the chamber during the mold filling stage of the VARTM process, and the other end is connected to an air compressor to provide positive pressure to the chamber during the mold filling and curing stages. The pressure chamber is equipped with a glass window for internal observation and a vacuum pressure gauge to reflect the air pressure in the chamber in real time.

During the resin injection stage, valves R1, R2, and R4 are opened and R3 is closed, to equalize the pressure inside and outside the vacuum bag, achieving a high vacuum of −0.92 bar. At this point, the fiber layer is relatively relaxed and the resin is rapidly injected into the vacuum bag at atmospheric pressure and converges at the outlet of the injection pipe. Once sufficient resin is injected, the vacuum pump is turned off, and valves R1 and R4 are closed, marking the end of the resin injection stage. The vacuum is maintained within the vacuum bag, while the pressure chamber is about to cancel the vacuum and fill with high pressure. During the filling and curing stages, valves R2 and R3 are opened and the air compressor is activated to obtain a high pressure in the chamber to enhance the resin diffusion and infiltration rate. Under the influence of pressure, the resin rapidly spreads towards the vacuum port and penetrates downward to infiltrate the fibers. After the filling is complete, the air compressor is deactivated and valves R2, R3, and R4 are closed while R1 is opened. High pressure is maintained inside the pressure chamber until the resin naturally cures. Excess resin flows back into the storage container through the injection pipe, preventing resin wastage.

### 3.2. Experiment Materials

The fiber reinforcement used for the preparation of the laminate specimens was SYT45-200g twill carbon fiber reinforcement produced by Zhongfu Shenying Carbon Fiber Corporation (Lianyungang, China), with a density of 1.8 g/cm^3^ and a superficial density of 200 g/m^2^. The matrix resin was a combination of 2511-1A/2511-1BM epoxy resin/curing agent produced by Shangwei Wind Power Materials Co. (Tianjin, China) with a mixing ratio of 10:3 and a viscosity of 0.25 Pa-s after mixing. The activation time was 1.5~2 h, and the density was 1.14 g/cm^3^ after curing. The distribution medium used was the PE-based mesh flow medium produced by Shanchuan Composite Materials Company (Qingdao, China). It has a maximum operating temperature of 120 °C, a surface density of 150 g/m^2^, and a thickness of 1 ± 0.1 mm.

### 3.3. Specimen Preparation

Carbon fiber composite laminates were fabricated using the conventional VARTM process and the enhanced VARTM process, with an aluminum alloy flat-bottom mold of 300 mm × 300 mm and a fiber reinforcement size of 250 mm × 250 mm, with a total of 8 layers laid at 0° or 90°. In order to keep the negative pressure gradient comparable to that of the VARTM process, positive pressures of 0.8 bar, 1.2 bar, and 1.6 bar were filled into the pressure vessel during the holding pressure curing stage of the enhanced VARTM process [22]. The blended resin was left to degas for 10 min, and all the prepared laminates were naturally cured at room temperature for 12 h and then sent to an electric blast drying oven, heated at 80 °C for 8 h. After completion, they were taken out of the molds, trimmed, and cut to obtain the composite laminate specimens.

A total of five sets of experimental samples were prepared: one set of the VARTM process, labeled as NDM (without distribution medium); one set of the VARTM process with distribution medium, labeled as DM (with distribution medium); and three sets of the enhanced VARTM process, labeled as 0.8 bar, 1.2 bar, and 1.6 bar.

The experimental samples were all prepared with the same type of carbon fiber, with very thin fiber reinforcement thicknesses and all with the same transverse permeability.

## 4. Experiment Results and Discussion

### 4.1. Filling Time

The change in resin quantity was recorded with an electronic scale and the mold filling process of the laminated panel was recorded with a high-speed video camera. The initiation of resin infiltration into the fiber layer is considered the starting point, and the moment when the square laminated panel is fully saturated marks the completion of the injection molding process. The time difference between the two events was recorded. The mold filling time of the enhanced VARTM process consists of two parts, the resin injection phase and the fiber infiltration phase. The mold filling times in the simulations are automatically calculated from the boundary conditions set by the simulation software COMSOL.

The simulation and experimental results of each group are shown in Table 1. The mold filling time of the VARTM process (NDM) is significantly longer than that of other experimental groups. This is primarily because the carbon fiber reinforcement is densely woven and pressed tightly under atmospheric pressure, resulting in a lack of sufficient resin flow channels, both between fiber bundles and between fiber layers. Liquid resin can only flow forward slowly under the influence of pressure differentials. In this experiment, the mold filling time for the specimen with an area of 250 mm × 250 mm prepared under NDM conditions was more than 1 h, which was at least an order of magnitude longer than that under other process conditions. However, the laminated panels produced under these conditions exhibited relatively good thickness and mechanical performance (as can be seen from the test results in the subsequent Section 4.2 and Section 4.3), mainly because the fibers were thoroughly and completely infiltrated under these conditions.

The VARTM process with the use of a distribution medium (DM) requires approximately 6 min for injection, making it the shortest injection molding time among all the experimental groups. This demonstrates that the distribution medium significantly enhances resin infiltration speed. However, it is important to note that the resin primarily flows rapidly across the surface of the distribution medium, and the underlying fiber layers are not immediately infiltrated. After passing through the distribution medium, the resin then begins to slowly penetrate downward under the influence of pressure differentials. This can result in a substantial number of air bubbles becoming trapped within the fiber layers. Therefore, after the mold filling process is completed, this process (DM) typically requires rinsing with resin for a period of time to expel air bubbles as much as possible.

In the enhanced VARTM process, the relative vacuum levels extracted in the first stage are all the same, at −0.92 bar. Therefore, the injection time for the samples under the three different external pressure conditions during the first stage is approximately the same and very brief, lasting only about 3 s. In the second stage, the increasing pressure within the pressure chamber facilitated the infiltration and mold filling of the resin among the relaxed fibers, resulting in a reduced time required for complete permeation of all fibers. It is evident that the mold filling time under 1.6 bar is reduced by approximately 30% compared to that under 0.8 bar.

Comparing the results of simulations and experiments, it is evident that all simulated mold filling times are shorter than the actual mold filling time, with a maximum error of 13.58%. There are two primary reasons for this discrepancy. Firstly, various interfering factors can arise during the experimental process, such as uneven fiber bundle structures, changes in resin viscosity with temperature and time, wrinkles in the vacuum bag, and partial blockage at the inlet and outlet. All these factors can lead to a longer actual mold filling time. Secondly, the model parameters and environmental conditions set in the simulation are often oversimplified and idealized, compared to the more complex experimental conditions, which naturally results in shorter mold filling time outcomes. Despite this discrepancy, it is evident from a combination of simulation and experimental results that there is a common trend among these different processes: as the external pressure increases, the time for resin mold filling decreases.

### 4.2. Dimensional Accuracy

**Figure 7:**
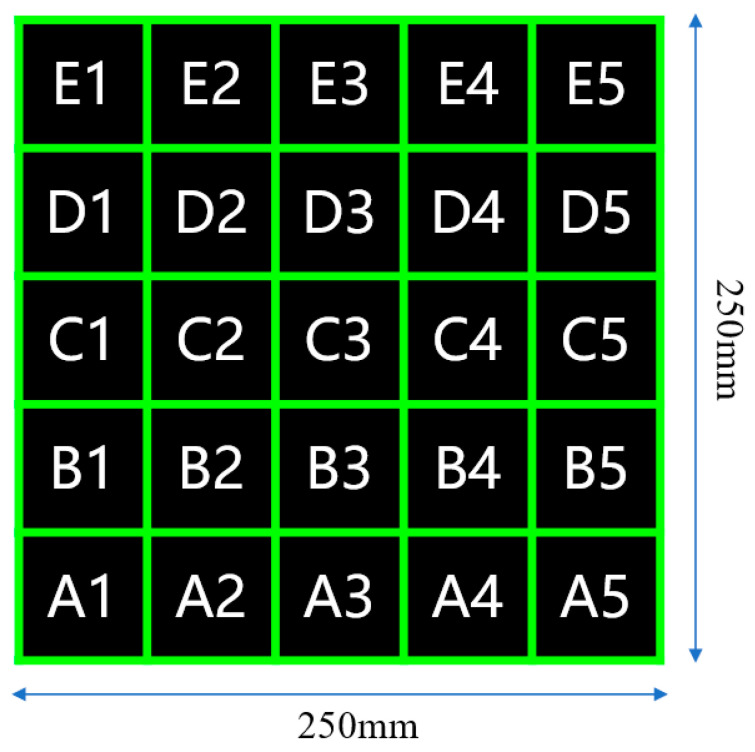
Zoning and numbering of composite panels (The letters in the figure represent rows, while the numbers represent columns).

**Figure 8:**
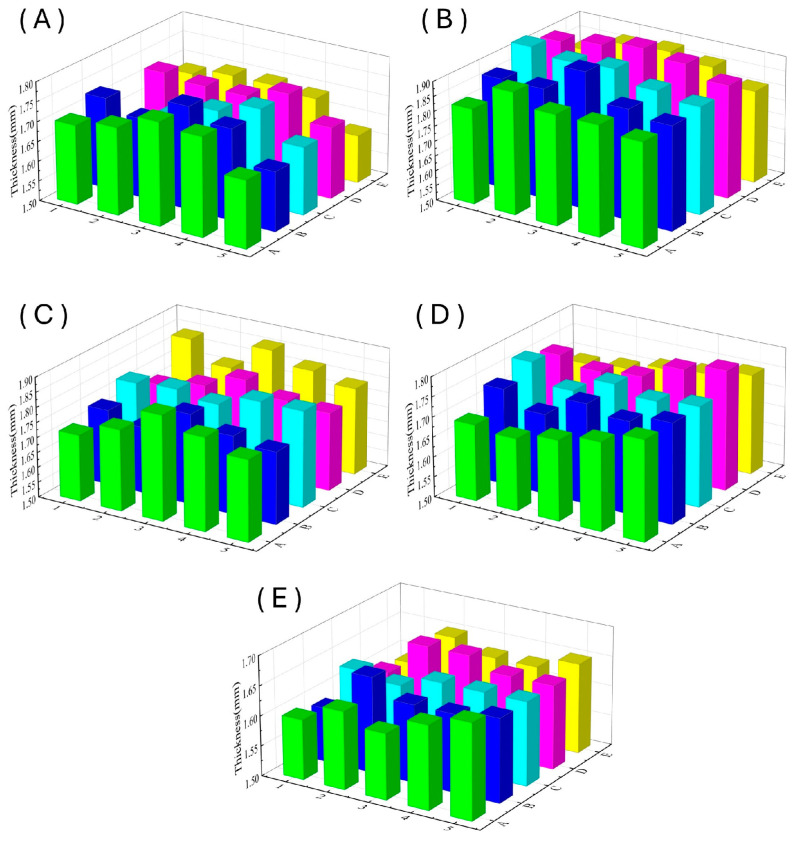
Laminate board thicknesses under different test conditions. (**A**) Blank group without distribution medium. (**B**) Blank group with distribution medium. (**C**) External compaction pressure of 0.8 bar. (**D**) External compaction pressure of 1.2 bar. (**E**) External compaction pressure of 1.6 bar.

The 250 mm × 250 mm composite laminate panel was divided into 5 × 5 grid, as shown in Figure 7. The thickness of each region was measured, with the resin injection port located at A3 and the vacuum vent at E3. The measurement results are shown in Figure 8. A three-dimensional column chart vividly demonstrates the thickness of each region of the laminate panel and its thickness uniformity.

**Figure 9:**
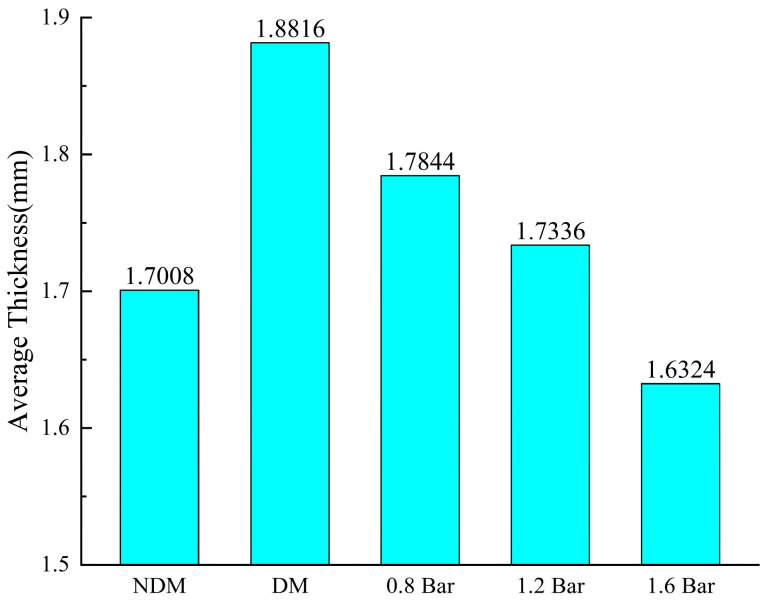
Average laminate thickness for each test condition.

Figure 9 illustrates the average thickness of the laminated panels under different experimental conditions. The laminated panels prepared using the VARTM process with the use of a distribution medium (DM) exhibit the maximum average thickness. This is because the fibers in the preform under the distribution medium have not been sufficiently compressed for a long duration, resulting in a large resin injection quantity. After resin infiltration, the fiber layers remain relatively relaxed, and excess resin cannot be expelled. Additionally, the distribution medium leads to increased surface roughness of the laminated panel, affecting thickness measurements to some extent [31].

The VARTM process (NDM) leads to thinner laminated panels due to its longer injection molding time, allowing for extended fiber compression and slower resin infiltration with a lower injection volume. This results in better surface quality and thinner thickness. The enhanced VARTM process maintains a consistent pressure during the natural curing process of the laminated panel, ensuring the extrusion of excess resin and bubbles before the curing of the resin. As the external pressure increases, the infiltration of the resin into the fibers during the mold filling and curing stages becomes more thorough and compact, resulting in a gradual decrease in the average thickness of the laminates. Under an external pressure condition of 1.6 bar, the average thickness of the laminated panel is already less than that of the laminated panel without a distribution medium, with a single-layer thickness of approximately 0.2 mm.

To more accurately quantify the dimensional accuracy of the laminated panels, the standard deviation was used to indicate the magnitude of thickness fluctuations among the different sample groups. And the following formulae were used to express the maximum deviation rate of laminated panels’ thickness to assess the acceptable level of thickness fluctuation.

(17) $$D_{x}=\left(h_{\max}-h_{\min}\right)/h_{\min}\times 100\%$$

where $h_{\max}$ and $h_{\min}$ denote the maximum and minimum values of plate thickness for each group of specimens, respectively.

The plate thickness data measured for each set of specimens were brought into the standard deviation calculation formula and Equation (17) to obtain the results shown in Table 2.

The standard deviation, represented by *S*, indicates that without applying external pressure, there is little difference in the uniformity of laminated panel thickness, and it is relatively poor across all conditions. When external pressure is applied, as the pressure increases, the uniformity of laminated panel thickness improves, and the dimensional accuracy becomes higher. The maximum deviation rate for all experimental groups is less than 10%, with the maximum deviation rate for the laminated panel thickness under 1.6 bar external pressure being 4.40%. This reduces the maximum deviation rate by more than half compared to the sample under no external pressure, proving an improvement in the quality of the laminate.

### 4.3. Mechanical Properties

**Figure 10:**
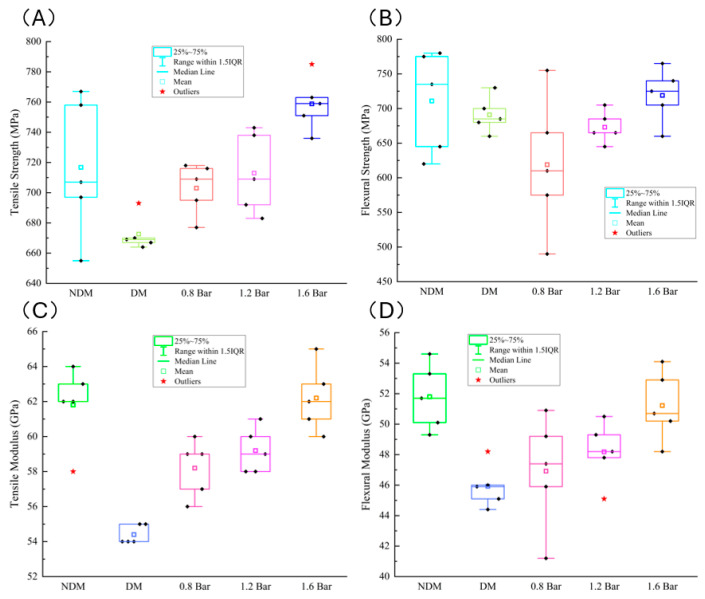
Mechanical properties of composite laminates. (**A**) Tensile strength. (**B**) Flexural strength. (**C**) Tensile modulus. (**D**) Flexural modulus.

The results of mechanical performance tests for the laminated panels are shown in Figure 10. Laminated panels produced using the VARTM process with the use of a distribution medium (DM) exhibit the lowest values for all performance parameters except flexural strength. However, at the same time, the results from the five samples are the most concentrated.

Laminated panels produced using the VARTM process (NDM) show relatively good performance parameters, comparable to those of laminated panels under an external pressure of 1.6 bar. However, there is a significant variation in the strength test results among the five samples, with a wide distribution range.

Under the enhanced VARTM process, the increase in pressure enhances the thoroughness and compactness of the resin infiltration into the fibers, resulting in a strong bond between them after curing. Consequently, the performance parameters of the laminates are improved. When the pressure reaches 1.6 bar, the average tensile strength of the laminated panels reaches around 760 MPa, and the average flexural strength is also approximately 720 MPa. The results from the five samples are relatively concentrated at this pressure level.

### 4.4. Void Content and Fiber Volume Fractions

Porosity and fiber volume fraction are important parameters that affect the mechanical properties of composite materials. The unsaturated flow of resin within the fiber layers is the fundamental cause of porosity. In this study, we measured the porosity of laminated panels under three different processes according to the ASTM D2734-16 [32] experimental standard.

(18) $$V=100-D_{c}\left(\frac{W_{m}}{D_{m}}+\frac{W_{f}}{D_{f}}\right)$$

where *V* denotes the void content of the composite; *D_c_* is the measured density of the composite (g/cm^3^); *W_m_* is the mass percentage of resin in the composite; *D_m_* is the density of resin (g/cm^3^); *W_f_* is the mass percentage of fiber in the composite; and *D_f_* is the density of fiber (g/cm^3^).

The parameters *W_m_* and *W_f_* mentioned above need to be obtained through experiments. The method used to measure the content of various components in the composite materials involves the pyrolysis decomposition of the resin matrix and follows the ASTM D2584-18 [33] standard for testing. Four samples of size 25 mm × 25 mm were cut from the laminated panel under each test condition. Additionally, a pure resin sample of size *Φ*10 × 20 mm was used as a control to measure the resin carbonization rate. These samples were placed in a muffle furnace and heated at a temperature of 565 °C for 0.5 h until the resin was completely carbonized. The change in mass before and after burning the samples was measured. The value from the sample with the largest deviation was discarded, and the average of the remaining three samples was calculated. The mass percentage of resin and fibers in the samples was calculated following the ASTM D3171-15 [34] test standard.

(19) $$W_{m}=\frac{M_{i}-M_{f}}{M_{i}\times \left(1-\frac{m_{d}}{m_{i}}\right)}\times 100$$

(20) $$W_{f}=\frac{M_{f}-M_{i}\times \frac{m_{d}}{m_{i}}}{M_{i}\times \left(1-\frac{m_{d}}{m_{i}}\right)}\times 100$$

where *M_i_* is the initial mass of the sample; *M_f_* is the residual mass of the sample after burning; *m_d_* is the residual mass of the pure resin sample after burning; and *m_i_* is the initial mass of the pure resin sample. The units are all g.

The carbonation rate of pure resin after burning is *m_d_*/*m_i_*; if the resin is decomposed into volatile substances after burning, then *m_d_* = 0, *M_f_* is the mass of the fibers in the sample, and the above Equations (19) and (20) are simplified as the following:

(21) $$W_{m}=\frac{M_{i}-M_{f}}{M_{i}}\times 100$$

(22) $$W_{f}=\frac{M_{f}}{M_{i}}\times 100$$

The fiber volume fraction was calculated using the following formula:

(23) $$V_{f}=W_{f}\times \frac{D_{c}}{D_{f}}=\frac{M_{f}-M_{i}\times \frac{m_{d}}{m_{i}}}{M_{i}\times \left(1-\frac{m_{d}}{m_{i}}\right)}\times 100\times \frac{D_{c}}{D_{f}}$$

**Figure 11:**
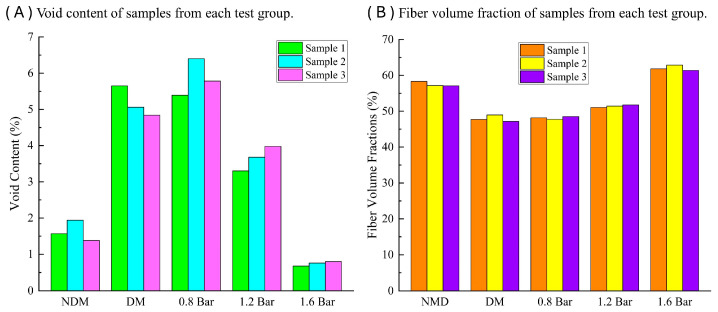
Void content and Fiber volume fraction of samples from each test group.

The experimental results for porosity and fiber volume fraction are shown in Figure 11. When the pressure reaches 1.6 bar, the porosity of the laminated panel is less than 1%, nearly equivalent to the porosity of the pre-impregnated product (prepreg) which is also less than 1% and can have a fiber volume fraction of 65% to 70%. Laminated panels without the use of a distribution medium have a porosity ranging from 1% to 2% [35], while the porosity of the remaining laminated panels is higher. Under an external pressure of 0.8 bar, the porosity of the laminated panel is higher than that of laminated panels without a distribution medium, and as the applied pressure increases, the porosity of the laminated panel decreases.

When the pressure reaches 1.6 bar, the fiber volume fraction of the laminated panel can reach 62%, slightly lower than the fiber volume fraction of prepreg products [2] but significantly higher than the fiber volume fraction of hand-laid products. Laminated panels without the use of a distribution medium have a fiber volume fraction slightly below 60%, while the fiber volume fraction of the remaining laminated panels is around 50%. With increasing applied pressure, the infiltration of the resin into the fibers becomes more thorough and compact, resulting in a higher fiber volume fraction and lower porosity of the laminates.

To further evaluate the fiber volume fraction of laminates with different thicknesses, we normalized the raw data obtained from the tests. Table 3 presents the normalized data, which effectively mitigated the confounding influence of thickness variations on the fiber volume fraction analysis. Notably, it can be observed that the laminate under an external pressure of 1.6 bar exhibited the highest fiber volume fraction per unit thickness, reaching 37.98%, which is higher than both the laminate without a flow medium (34.47%) and the laminate with a flow medium (26.04%).

As the applied pressure increases, the porosity of the laminate decreases and the fiber volume fraction increases, almost reaching the level of pre-impregnated products, so that the mechanical properties of the laminate become better with the increase in the applied pressure.

In this paper, we utilized simulations to validate the permeation behavior of resin within and between multiple layers of fibers under the influence of pressure fields, investigating the flow patterns, permeation velocities, and volume fractions of resin during the molding process. We identified consistent patterns which can effectively guide the conduct of experiments. The molding time observed in experimental results correlates closely with simulated molding time. Additionally, experimental findings provide valuable supplementary explanations for parameters such as laminate thickness, mechanical properties, porosity, and fiber volume fraction, which are not directly observable in simulations. The combination of insights from simulations and experimental results can validate the beneficial impact of the externally applied pressure field in the enhanced VARTM process on laminate performance.

## 5. Conclusions

This paper proposes an enhanced VARTM process, which features a bi-directional adjustable pressure unit for vacuuming or pressurizing the molding process. This constitutes the primary innovation of the study, aimed at enhancing the performance of molded composite components. Theoretical analysis and simulation results indicate that under different applied pressure conditions, the resin flow front morphology within and between fiber layers is almost identical. The higher the applied pressure, the faster the resin flow, and meanwhile the lower the resin volume fraction. The resin infiltration within the top fiber layer is extremely fast, and the transverse infiltration in the thickness direction is slower. A certain angle is formed between the resin flow front and the horizontal plane within each layer, a folded-line-shaped resin flow front is formed, and the angle gradually increases when the pressure is higher and the flow front is farther away. For typical thin-walled components, the macroscopic flow patterns of the resin under different pressures are nearly identical. The flow front initially infiltrates and spreads forward in an arc shape, then in an arc shape resembling a bow, and the curvature of the flow front continuously decreases with increasing penetration distance. Ultimately, it approaches a straight line near the vacuum vent. External pressure enhances the infiltration rate, and the bubbles formed by unsaturated flow are gradually expelled during the pressure-maintaining phase. This leads to an increase in fiber volume fraction and a decrease in porosity.

Experiments were carried out to validate the resin flow patterns and advantages of the enhanced VARTM process. Compared to the two scenarios of the regular VARTM process with and without a distribution medium, the infusion efficiency, thickness uniformity, mechanical performance, porosity, and fiber volume fraction are all promoted with the enhanced VARTM process. The results indicate that the composite’s mechanical performance and fiber volume fraction increase with increasing pressure, while thickness and porosity decrease correspondingly. Under an external pressure of 1.6 bar, the laminated panel exhibits an average tensile strength of around 760 MPa, an average flexural strength of approximately 720 MPa, a fiber volume fraction as high as 62%, and a porosity below 1%. Meanwhile, the infusion efficiency is promoted and the material preparation time can be greatly reduced.

Through theoretical analysis and simulation methods, this study has revealed the three-dimensional infiltration characteristics of resin in multi-layer media and the planar infiltration characteristics in thin-walled components. It has also elucidated the control mechanisms of fiber volume fraction and porosity under external pressure. By comparing the experimental results of the enhanced VARTM process with those of the regular VARTM process, the experimental data have validated the accuracy of the models and mechanisms, as well as the superiority of the proposed enhanced VARTM process. This comprehensive research, ranging from theory, simulation, to experimentation, provides a realistic case study for the improvement and development of the VARTM process. Additionally, it demonstrates the applicability of the simulation and experimental conditions, satisfying the scalability requirements for practical industrial applications. The study also exhibits certain limitations and shortcomings. Firstly, the scope of pressure investigation was limited, encompassing experiments and discussions solely at pressures of 0.8 bar, 1.2 bar, and 1.6 bar. Future research could encompass a broader range of pressure testing to derive more universally applicable principles and optimize experimental parameters further. Furthermore, our study solely focused on samples with simple planar geometries. Therefore, additional exploration and research are necessary to apply this optimization approach to molding processes involving complex curved surfaces or composite structural components.

## Figures and Tables

**Table 1:** Time required for mold filling under different test conditions.

Test Conditions	Time Required for Actual Mold Filling [s]	Mold Filling Time Calculated by Simulation [s]
NDM	3724	—
DM	355	—
0.8 Bar	559	525
1.2 Bar	485	420
1.6 Bar	408	350

**Table 2:** Standard deviations of laminated panel thickness at various locations under different experimental conditions.

Test Conditions	Value of Standard Deviation *S* [/1000 mm]	Maximum Deviation Rate *D_x_* [%]
NDM	38.15	9.32
DM	37.28	8.29
0.8 bar	37.85	8.14
1.2 bar	28.97	7.14
1.6 bar	20.45	4.40

**Table 3:** Raw data and normalized form of fiber volume fraction under different experimental conditions.

Test Conditions	Raw Data of Fiber Volume Fraction [%]	Normalized Form of Fiber Volume Fraction [%]
NDM	58.63	34.47
DM	48.99	26.04
0.8 bar	48.45	27.15
1.2 bar	51.87	29.92
1.6 bar	62.03	37.98

## Data Availability

The raw/processed data required to reproduce these findings will be made available on request.

## References

[1] Matsuzaki R., Kobayashi S., Todoroki A., Mizutani Y. (2011). Control of resin flow/temperature using multifunctional interdigital electrode array film during a VaRTM process. Compos. Part A Appl. Sci. Manuf..

[2] Yalcinkaya M.A., Sozer E.M., Altan M.C. (2017). Fabrication of high quality composite laminates by pressurized and heated-VARTM. Compos. Part A Appl. Sci. Manuf..

[3] Fu X., Zhang C., Liang R., Wang B., Fielding J.C. (2011). High Temperature Vacuum Assisted Resin Transfer Molding of Phenylethynyl Terminated Imide Composites. Polym. Compos..

[4] Bhunia S., Niyogi D., Marru P., Neogi S. (2014). Modelling of Curing Kinetics of Amine Cured Epoxy Resins for Vacuum Assisted Resin Infusion Molding. Can. J. Chem. Eng..

[5] Wu D., Larsson R., Blinzler B. (2021). A preform deformation and resin flow coupled model including the cure kinetics and chemo-rheology for the VARTM process. Int. J. Mater. Form..

[6] Zhang K., Gu Y., Zhang J., Li M., Wang S., Zhang Z. (2016). Rapid curing vacuum-assisted resin infusion molding using silicone rubber sheet heater and the effect of cooling process on the properties of carbon fiber/epoxy composites. J. Compos. Mater..

[7] Allende M., Mohan R.V., Walsh S.M. (2004). Experimental and numerical analysis of flow behavior in the FASTRAC liquid composite manufacturing process. Polym. Compos..

[8] Lee H., Jung K., Park H. (2021). Study on Structural Design and Analysis of Composite Boat Hull Manufactured by Resin Infusion Simulation. Materials.

[9] Ricciardi M.R., Antonucci V., Durante M., Giordano M., Nele L., Starace G., Langella A. (2014). A new cost-saving vacuum infusion process for fiber-reinforced composites: Pulsed infusion. J. Compos. Mater..

[10] Antonucci V., Giordano M., Nele L., Langella A., Durante M., Ricciardi M.R., Starace G. (2009). Sistema di Fabbricazione di Materiali Compositi con Infusione Pulsata (Pulsed Infusion). Italian Pending Patent.

[11] Ricciardi M.R., Martone A., Borriello A., Zarrelli M., Giordano M., Langella A., Antonucci V. (2017). Mechanical behavior of hybrid fiber-reinforced composites manufactured by pulse infusion. Polym. Compos..

[12] Alam Khan L., Mahmood A.H., Ahmed S., Day R.J. (2013). Effect of double vacuum bagging (DVB) in quickstep processing on the properties of 977-2A carbon/epoxy composites. Polym. Compos..

[13] Liu Y.-N., Yuan C., Liu C., Pan J., Dong Q. (2019). Study on the resin infusion process based on automated fiber placement fabricated dry fiber preform. Sci. Rep..

[14] Alms J., Advani S.G. (2007). Simulation and experimental validation of flow flooding chamber method of resin delivery in liquid composite molding. Compos. Part A Appl. Sci. Manuf..

[15] Alms J.B., Advani S.G., Glancey J.L. (2011). Liquid Composite Molding control methodologies using Vacuum Induced Preform Relaxation. Compos. Part A Appl. Sci. Manuf..

[16] Alms J.B., Glancey J.L., Advani S.G. (2010). Mechanical properties of composite structures fabricated with the vacuum induced preform relaxation process. Compos. Struct..

[17] Chen D., Arakawa K., Uchino M. (2016). Effects of the addition of a cover mold on resin flow and the quality of the finished product in vacuum-assisted resin transfer molding. Polym. Compos..

[18] Yalcinkaya M.A., Sozer E.M., Altan M.C. (2019). Effect of external pressure and resin flushing on reduction of process-induced voids and enhancement of laminate quality in heated-VARTM. Compos. Part A Appl. Sci. Manuf..

[19] Yalcinkaya M.A., Sozer E.M., Altan M.C. (2019). Dynamic pressure control in VARTM: Rapid fabrication of laminates with high fiber volume fraction and improved dimensional uniformity. Polym. Compos..

[20] Rubino F., Carlone P. (2019). A Semi-Analytical Model to Predict Infusion Time and Reinforcement Thickness in VARTM and SCRIMP Processes. Polymers.

[21] Sun X., Li S., Lee L.J. (1998). Mold filling analysis in vacuum-assisted resin transfer molding. Part I: SCRIMP based on a high-permeable medium. Polym. Compos..

[22] Kuentzer N., Simacek P., Advani S.G., Walsh S. (2007). Correlation of void distribution to VARTM manufacturing techniques. Compos. Part A Appl. Sci. Manuf..

[23] Ravey C., Ruiz E., Trochu F. (2014). Determination of the optimal impregnation velocity in Resin Transfer Molding by capillary rise experiments and infrared thermography. Compos. Sci. Technol..

[24] Yenilmez B., Senan M., Sozer E.M. (2009). Variation of part thickness and compaction pressure in vacuum infusion process. Compos. Sci. Technol..

[25] Sakin R. (2021). Layup Design Optimization for E-glass Woven Roving Fabric Reinforced Polyester Composite Laminates Produced by VARTM. Fibers Polym..

[26] Becker D., Mitschang P. (2015). Influence of preforming technology on the out-of-plane impregnation behavior of textiles. Compos. Part A Appl. Sci. Manuf..

[27] Rimmel O., Becker D., Mitschang P. (2016). Maximizing the out-of-plane-permeability of preforms manufactured by dry fiber placement. Adv. Manuf. Polym. Compos. Sci..

[28] Aziz A., Ali M., Zeng X., Umer R., Schubel P., Cantwell W. (2017). Transverse permeability of dry fiber preforms manufactured by automated fiber placement. Compos. Sci. Technol..

[29] Liu X., Li Y., Zhu J., Wang Y., Qing X. (2021). Monitoring of resin flow front and degree of cure in vacuum-assisted resin infusion process using multifunctional piezoelectric sensor network. Polym. Compos..

[30] Michaud V. (2016). A Review of Non-saturated Resin Flow in Liquid Composite Moulding processes. Transp. Porous Media.

[31] Vilà J., Sket F., Wilde F., Requena G., González C., Llorca J. (2015). An in situ investigation of microscopic infusion and void transport during vacuum-assisted infiltration by means of X-ray computed tomography. Compos. Sci. Technol..

[32] (2016). Standard Test Methods for Void Content of Reinforced Plastics.

[33] (2018). Standard Test Methods for Ignition Loss of Cured Reinforced Resins.

[34] (2015). Standard Test Methods for Constituent Content of Composite Materials.

[35] Amirkhosravi M., Pishvar M., Altan M.C. (2017). Improving laminate quality in wet lay-up/vacuum bag processes by magnet assisted composite manufacturing (MACM). Compos. Part A Appl. Sci. Manuf..

